# Improving wear time compliance with a 24-hour waist-worn accelerometer protocol in the International Study of Childhood Obesity, Lifestyle and the Environment (ISCOLE)

**DOI:** 10.1186/s12966-015-0172-x

**Published:** 2015-02-11

**Authors:** Catrine Tudor-Locke, Tiago V Barreira, John M Schuna, Emily F Mire, Jean-Philippe Chaput, Mikael Fogelholm, Gang Hu, Rebecca Kuriyan, Anura Kurpad, Estelle V Lambert, Carol Maher, José Maia, Victor Matsudo, Tim Olds, Vincent Onywera, Olga L Sarmiento, Martyn Standage, Mark S Tremblay, Pei Zhao, Timothy S Church, Peter T Katzmarzyk, for the ISCOLE Research Group

**Affiliations:** Pennington Biomedical Research Center, 6400 Perkins Road, Baton Rouge, LA 70808 USA; Syracuse University, Syracuse, USA; Oregon State University, Corvallis, USA; Children’s Hospital of Eastern Ontario Research Institute, Ottawa, Canada; University of Helsinki, Helsinki, Finland; St. Johns Research Institute, Bangalore, India; University of Cape Town, Cape Town, South Africa; University of South Australia, Adelaide, Australia; CIFI2D, Faculdade de Desporto, University of Porto, Porto, Portugal; Center of Studies of the Physical Fitness Research Laboratory from Sao Caetano do Sul (CELAFISCS), Sao Paulo, Brazil; Kenyatta University, Nairobi, Kenya; School of Medicine, Universidad de los Andes, Bogota, Colombia; University of Bath, Bath, UK; Tianjin Women’s and Children’s Health Center, Tianjin, China

**Keywords:** Accelerometry, Measurement, Physical activity, Exercise, Sedentary time

## Abstract

**Background:**

We compared 24-hour waist-worn accelerometer wear time characteristics of 9–11 year old children in the International Study of Childhood Obesity, Lifestyle and the Environment (ISCOLE) to similarly aged U.S. children providing waking-hours waist-worn accelerometer data in the 2003–2006 National Health and Nutrition Examination Survey (NHANES).

**Methods:**

Valid cases were defined as having ≥4 days with ≥10 hours of waking wear time in a 24-hour period, including one weekend day. Previously published algorithms for extracting total sleep episode time from 24-hour accelerometer data and for identifying wear time (in both the 24-hour and waking-hours protocols) were applied. The number of valid days obtained and a ratio (percent) of valid cases to the number of participants originally wearing an accelerometer were computed for both ISCOLE and NHANES. Given the two surveys’ discrepant sampling designs, wear time (minutes/day, hours/day) from U.S. ISCOLE was compared to NHANES using a meta-analytic approach. Wear time for the 11 additional countries participating in ISCOLE were graphically compared with NHANES.

**Results:**

491 U.S. ISCOLE children (9.92±0.03 years of age [M±SE]) and 586 NHANES children (10.43 ± 0.04 years of age) were deemed valid cases. The ratio of valid cases to the number of participants originally wearing an accelerometer was 76.7% in U.S. ISCOLE and 62.6% in NHANES. Wear time averaged 1357.0 ± 4.2 minutes per 24-hour day in ISCOLE. Waking wear time was 884.4 ± 2.2 minutes/day for U.S. ISCOLE children and 822.6 ± 4.3 minutes/day in NHANES children (difference = 61.8 minutes/day, *p* < 0.001). Wear time characteristics were consistently higher in all ISCOLE study sites compared to the NHANES protocol.

**Conclusions:**

A 24-hour waist-worn accelerometry protocol implemented in U.S. children produced 22.6 out of 24 hours of possible wear time, and 61.8 more minutes/day of waking wear time than a similarly implemented and processed waking wear time waist-worn accelerometry protocol. Consistent results were obtained internationally. The 24-hour protocol may produce an important increase in wear time compliance that also provides an opportunity to study the total sleep episode time separate and distinct from physical activity and sedentary time detected during waking-hours.

**Trial registration:**

ClinicalTrials.gov NCT01722500.

**Electronic supplementary material:**

The online version of this article (doi:10.1186/s12966-015-0172-x) contains supplementary material, which is available to authorized users.

## Background

The International Study of Childhood Obesity, Lifestyle and the Environment (ISCOLE) [1] is a multi-national cross-sectional study of lifestyle and environmental factors that may influence children’s obesity. Data were collected from over 500 children (targeting a mean age of 10 years) from sites in each of the following 12 countries: Australia, Brazil, Canada, China, Colombia, Finland, India, Kenya, Portugal, South Africa, the United Kingdom, and the United States of America. One of the key data points collected across all countries was objectively monitored physical activity using the waist-worn ActiGraph GT3X+ accelerometer (ActiGraph LLC, Pensacola, FL, USA). This instrument provides manifold amounts of time-stamped data that can be analyzed in numerous ways to represent free-living movement (and non-movement) events and patterns. Valid estimates of objectively monitored physical activity and sedentary time using accelerometry during waking-hours are threatened by insufficient wear time compliance [2-4]; thus, the pursuit of methodological improvements is warranted. Some studies are currently pursuing a 24-hour accelerometer protocol while also switching the accelerometer attachment site to the wrist [5]; however, those data are not yet available. Unfortunately, concurrent implementation of these two protocol changes will ultimately obscure conclusions about their separate effects on wear time [6]. In ISCOLE, a 24-hour accelerometer protocol was selected in an attempt to increase wear time compliance while maintaining the more typical waist attachment site [1].

The 2003–2006 cycles of the National Health and Nutrition Examination Survey (NHANES) collected waist-worn accelerometer (AM-7164, ActiGraph, LLC, Pensacola, FL, USA) data on a subsample of participants that included children. In contrast to ISCOLE, NHANES employed a waking-hours protocol. The purpose of this paper is to describe the multiple-day 24-hour waist-worn accelerometer protocol implemented with 9–11 year old children participating in ISCOLE and to compare wear time characteristics to similarly aged U.S. children following a multiple-day waking-hours only protocol during the 2003–2006 NHANES accelerometer substudies. Although we provide wear time data across all countries, we focus the NHANES comparison on ISCOLE data collected in the U.S.

## Methods

### ISCOLE vs. 2003–2006 NHANES

The ISCOLE protocol was approved by the Pennington Biomedical Research Center Institutional Review Board and local ethics approvals were also obtained in each participating country. Written informed parental consent and child assent (in those study sites where it was required) were obtained prior to collection of any study data. The National Center for Health Statistics ethics review board approved the original NHANES survey protocols, and informed consent was obtained for all NHANES participants. Data from the 2003–2004 NHANES and the 2005–2006 NHANES were downloaded from the Centers for Disease Control and Prevention website (ftp://ftp.cdc.gov/pub/Health_Statistics/nchs/nhanes/2003-2004/PAXRAW_C.EXE and ftp://ftp.cdc.gov/pub/Health_Statistics/nchs/nhanes/2005-2006/PAXRAW_D.EXE) and combined to maximize the size of the comparative sample. Since the ISCOLE data set focused on children 9–11 years of age, we isolated only those 2003–2006 NHANES accelerometer data for similarly-aged children. Comparable descriptive characteristics for both U.S.-based data sets included sample size, age, BMI, % overweight and obese, a count of valid days, and wear times (minutes/day, hours/day).

The design and methods of the ISCOLE study have been previously presented [1], however, only a general description of the accelerometry protocol was provided. A detailed manual describing the accelerometer data collection methods, including the management and treatment of the data is presented in Additional file 1. The design and methods of the 2003–2006 NHANES accelerometer data collection is available publically on-line at (http://www.cdc.gov/nchs/data/nhanes/nhanes_05_06/BM.pdf) and http://www.cdc.gov/nchs/nhanes/nhanes2003-2004/PAXRAW_C.htm) and have been presented previously in numerous publications that have been catalogued elsewhere [7]. Table 1 presents a summary comparison of accelerometer data collection and data management factors for both ISCOLE and the 2003–2006 NHANES. Most pertinent to this analysis, ISCOLE implemented a 24-hour wear time protocol, instructing children to remove the accelerometer only for water-based activities (e.g., bathing or swimming, although the GT3X+ is a water proof device). In contrast, NHANES instructed participants to remove the accelerometer for water-based activities *and* at bed time and to re-attach it in the morning upon awakening. Both protocols called for 7 consecutive days of wear. The U.S. ISCOLE site chose to employ all of the compliance enhancing strategies catalogued in Table 1, including phone calls, daily visits to school, and distribution of small daily incentives (e.g., erasers, stickers). Although all other ISCOLE country sites placed phone calls to parents, additional compliance enhancing strategies (incentives, daily visits to schools, etc.) were ultimately shaped by the site Principal Investigator considering local customs, conventions, opportunities and tolerances. These were not explicitly tracked outside of the U.S. site.

**Table 1:** **Comparison of ISCOLE and 2003–2006 NHANES on accelerometer data collection and data management factors**

	**ISCOLE (2012–2013)**	**NHANES 2003-2006**
**Data collection**		
Instrument	Actigraph GT3X+	Actigraph 7164
Instrument initialization	Started data collection at midnight of the day the device was received, data collected at 80 Hz.	Started data collection when device was received, data collected at 60 sec epochs.
Instrument wear regimen	Worn at mid-axillary line, lying on the iliac crest for 24 hours, removing while bathing and swimming (even though GT3X+ is a waterproof device).	Worn at mid-axillary line, lying on the iliac crest during wake time, removing while bathing and swimming.
Instrument wear instructions	Instructions were read and a hard-copy paper was handed out.	Instructions were read and a hard-copy paper was handed out.
Compliance enhancing strategies	In U.S. ISCOLE: Phone calls, daily visits to school, small daily incentives (e.g., erasers, stickers).	Monetary incentive, available by phone to answer questions or concerns, reminder postcard for return mailing of instrument.
Instrument return	Research staff collected the devices from the children.	Instruments were mailed back to NHANES warehouse.
Data download	Raw data downloaded and processed simultaneously. Processed data were in 1 sec epoch with the low frequency filter and included the 3 axis of orientation, steps, lux, and inclinometer.	Processed data in 60 sec epochs contained activity counts in the x-axis and steps.
Immediate determination of valid data	Using a simple algorithm, data checked to include ≥ 4 days, including 1 weekend day with > 10 h/day of wear time	Only checked whether downloaded or not downloaded
Participant checklist	Used to track instruments’ serial numbers, distribution and return dates, as well as record compliance checks.	Computerized management system
Data transfer	Data were uploaded to a secure website to be retrieved by the coordinating center	Not necessary
**Data management**		
Visual quality control check	File names and file sizes were checked	Screened for possible outliers and code data as reliable or questionable
SAS dataset creation	Files were re-integrated to 60 sec epochs and combined into a country specific dataset	Details not available
Automated quality control checks	Using an automated process, instrument serial number, distribution and return dates were checked against the participant checklist	Details not available
Final dataset	Once data checks were completed 3 datasets were created, 60 sec epoch with low frequency extension, 60 sec epoch with regular filter, and 15 sec epoch with low frequency extension.	Details not available
Data organization	Date, time, sequence of data and day of the week variables were created.	Date, time, sequence of data and day of the week variables were created.
	Additional variables (e.g., vector magnitude) were created and processes (e.g., separating date and time) were undertaken	
Initialization errors	Verified devices were initialized midnight of the first day	Details not available
Additional cleaning	The last day of data was deleted as well as any day after 7 days of data collection	Details not available
Finalization	Once all queries were resolved sequential 24-hour days were identified and labeled for midnight-to-midnight and noon-to-noon analysis	Details not available

N.B. Details for ISCOLE data collection and data management presented in Additional file 1. Details for NHANES data collections located at http://www.cdc.gov/nchs/data/nhanes/nhanes_05_06/BM.pdf.

Details for NHANES data management located at http://www.cdc.gov/nchs/nhanes/nhanes2003-2004/PAXRAW_C.htm.

### Data treatment

As per an *a priori* decision made during ISCOLE protocol planning (see Additional file 1), all data sets were reduced to those children with ≥ 4 days with at least 10 hours of waking wear time in a 24-hour period, including one weekend day. We further reduced the NHANES data set, culling those cases with flags denoting accelerometer “out of calibration” or “unreliable” data [8].

Treatment of the 24-hour data obtained from ISCOLE participants required that we first identify the nocturnal sleep period time (time of sleep onset to the end of sleep, including all sleep epochs and wakefulness after onset [9]) before considering any residual non-wear time, and thus wear time. Sleep period time for each participant was determined using a novel and fully-automated algorithm specifically developed for use in ISCOLE and other epidemiological studies employing a 24-hour waist-worn accelerometer protocol in children [10]. This algorithm produced sleep period time estimates similar to those obtained with expert visual inspection of accelerometer data [10]. The algorithm has been further refined to focus on nocturnal sleep and to account for episodes of wakefulness during sleep period time, so that total sleep episode time (TSET) could be calculated [11]. This count of minutes from all nocturnal episodes of sleep were considered non-waking minutes.

A separate non-wear algorithm was run on all remaining minutes not identified as part of the TSET in previous data processing in all ISCOLE data sets. Non-wear periods were defined as any sequence of at least 20 consecutive minutes of 0 activity counts [12]. Remaining epochs in the minute-by-minute ISCOLE accelerometry data file not identified as part of the TSET or non-wear were labeled as waking wear time. The same algorithmic process (≥20 consecutive minutes of 0 activity counts) was applied to the NHANES data to determine non-wear time, and subsequently calculate wear time as the difference between 24 hours and identified non-wear time.

### Statistical analysis

Descriptive characteristics were calculated for the total sample and by sex as means (± SE) or frequencies as appropriate for each of the two U.S. based (ISCOLE and NHANES) data sets. Descriptive characteristics for the 11 additional ISCOLE country sites focused solely on sample size and wear time estimates (mean and 95% CI). Specifically, total wear time logged over the 24-hour protocol was computed for each ISCOLE country’s data set; a similar variable could not be computed for the NHANES waking-hours protocol. Waking wear time (minutes/day, hours/day) for valid cases in the ISCOLE data sets represented children with at least 4 days and at least 10 hours of waking wear time in a 24-hour period, including one weekend day. The comparable variable for valid cases in the 2003–2006 NHANES data set represented children with at least 4 days and at least 10 hours of wear time over a waking-hours monitoring period, including one weekend day.

We report the ratio (percent) of valid cases to the number of participants originally wearing an accelerometer for these two U.S. based data sets. We compared age, BMI, % overweight and obese, number of valid days, and waking wear time estimates from the U.S. ISCOLE site to similar NHANES data. Comparisons (U.S. ISCOLE vs. NHANES) were performed for the entire sample (boys and girls combined), and separately for boys and girls using Cochran’s Q-Test for Heterogeneity evaluated at 1 degree of freedom. All NHANES estimates were produced using procedures for sample survey data, while incorporating appropriate sampling weights, to account for the complex, multistage probability design of NHANES. Waking wear time for each of the 11 additional countries participating in ISCOLE was graphically (mean and 95% CI) compared with NHANES wear time.

## Results

Accelerometers were worn by 648 unique, age-eligible participants at the U.S. ISCOLE site (17 participants were asked to wear the device twice to obtain sufficient amounts of data not evident during the first wearing period), and these data were reduced for this analysis to 491 cases with a sufficient number of valid days (including 15 cases corresponding to participants who wore the device a second time to satisfy compliance requirements). We conducted sensitivity analyses to determine the effect of including/excluding data from the 15 participants who wore the device a second time. No substantive difference in mean estimates were observed and conclusions for hypothesis tests were identical for the full (n = 491) and reduced samples (n = 476). As such, all analyses presented herein utilized the full U.S. ISCOLE sample (n = 491). The 2003–2004 NHANES contained 452 accelerometer files and the 2005–2006 NHANES contained 535 files. Together, the 2003–2006 NHANES data set yielded 586 cases after including only those 618 with a sufficient number of valid days and after further eliminating 29 participants with accelerometers labeled as “out of calibration” and another 3 with noted “unreliable” data. The ratio of valid cases to the number of participants originally wearing an accelerometer was 491/640 (76.7%) in U.S. ISCOLE and 618/987 (62.6%) in NHANES.

**Figure 1:**
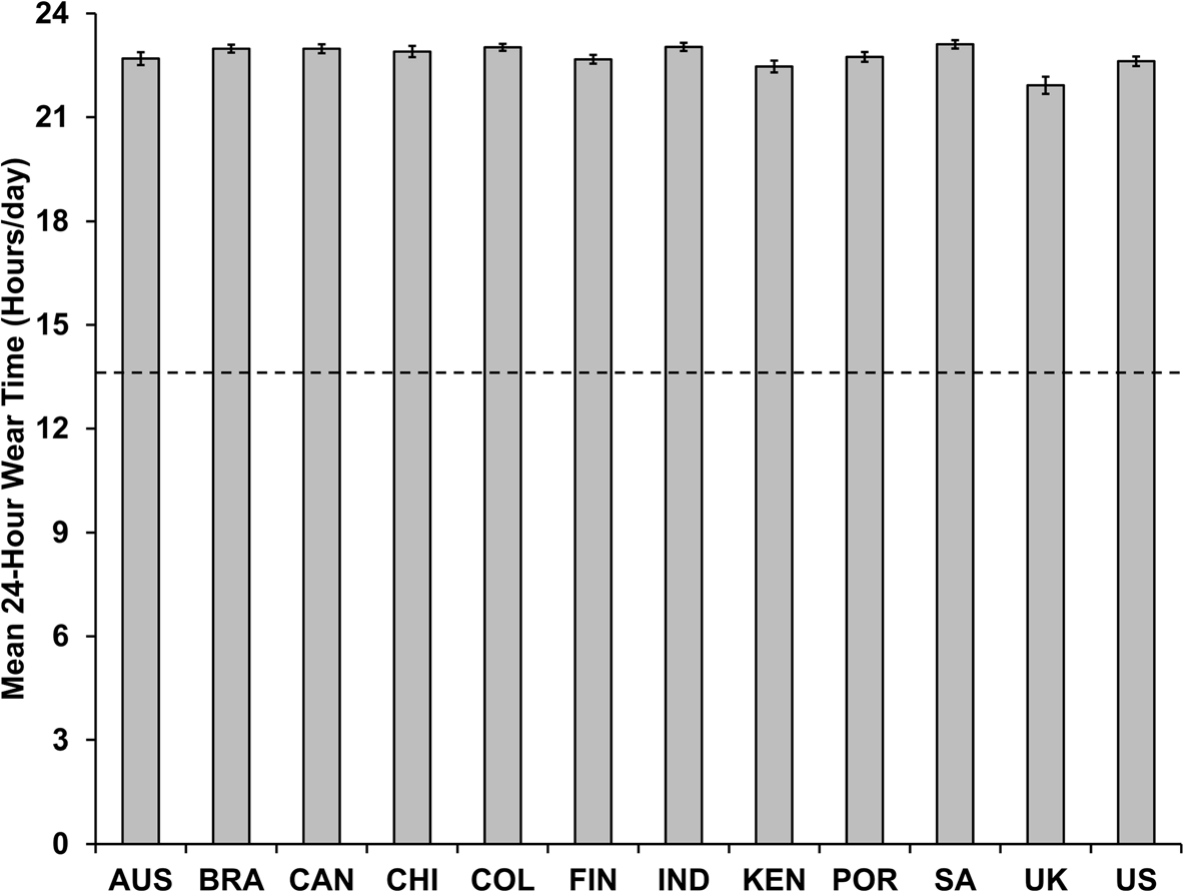
**Mean 24-hour wear time (and 95% CI) recorded in 9–11 year old children from 12 ISCOLE country study sites employing a 24-hour accelerometer protocol relative to children of the same age range from 2003–2006 NHANES employing a waking-hours accelerometer protocol.** Dashed line represents mean wear time (13.7 hours/day) for 9–11 year old children from 2003–2006 NHANES. AUS = Australia; BRA = Brazil, CAN = Canada; CHI = China; COL = Columbia; FIN = Finland; IND = India; KEN = Kenya; POR = Portugal; SA = South Africa; UK = United Kingdom; US = United States.

**Figure 2:**
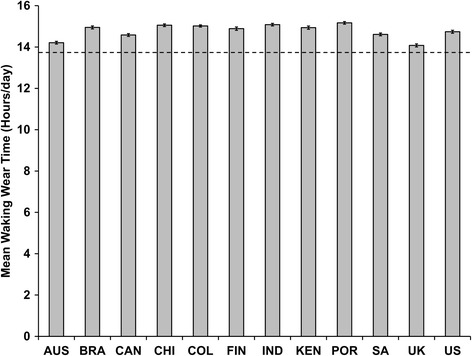
**Mean waking wear time (and 95% CI) recorded in 9–11 year old children from 12 ISCOLE country study sites employing a 24-hour accelerometer protocol relative to children of the same age range from 2003–2006 NHANES employing a waking-hours accelerometer protocol.** Dashed line represents mean wear time (13.7 hours/day) for 9–11 year old children from 2003–2006 NHANES. AUS = Australia; BRA = Brazil, CAN = Canada; CHI = China; COL = Columbia; FIN = Finland; IND = India; KEN = Kenya; POR = Portugal; SA = South Africa; UK = United Kingdom; US = United States.

Descriptive characteristics and statistical comparisons between U.S. ISCOLE and NHANES are presented in Table 2. U.S. ISCOLE averaged 6.4 valid days whereas NHANES averaged 6.0 valid days (*p* < 0.001). The U.S. ISCOLE sample averaged 1357.0 ± 4.2 minutes/day of wear time over the total possible 24-hour protocol asked of the participating children. Since the NHANES used a waking-hours protocol, a directly comparable variable is not possible. Estimates of waking wear time are directly comparable, however. U.S. ISCOLE children averaged 884.4 ± 2.2 minutes/day and NHANES children averaged 822.6 ± 4.3 minutes/day, representing a difference of 61.8 minutes in favor of the 24-hour protocol (*p* < 0.001). Graphical presentations of mean 24-hour wear time and mean waking wear time (Figures 1 and 2, respectively, both expressed in hours) for each of the ISCOLE country study sites are contrasted against mean wear time derived from 2003–2006 NHANES.

**Table 2:** **Descriptive characteristics of 9–11 year old U.S. children participating in accelerometer data collection during the 2012–2013 ISCOLE study and in the 2003–2006 NHANES accelerometer sub-study**

	**Boys**				**Girls**				**Total**			
	**U.S. ISCOLE**	**NHANES**	**Q**	^**†**^ ***p***	**U.S. ISCOLE**	**NHANES**	**Q**	^**†**^ ***p***	**U.S. ISCOLE**	**NHANES**	**Q**	^**†**^ ***p***
**N**	203	285	-	-	288	301	-	-	491	586	-	-
**Age (years)**	10.03 ± 0.04	10.42 ± 0.06	29.25	<0.001	9.85 ± 0.03	10.45 ± 0.05	105.88	<0.001	9.92 ± 0.03	10.43 ± 0.04	104.04	<0.001
**BMI (kg/m** ^**2**^ )*	18.65 ± 0.25	19.11 ± 0.30	1.39	0.239	19.04 ± 0.24	19.97 ± 0.39	4.12	0.042	18.87 ± 0.18	19.52 ± 0.24	4.41	0.036
**Overweight (%)**	14.3	15.0	0.08	0.779	18.4	18.9	0.02	0.880	16.7	16.8	0.01	0.960
**Obese (%)**	16.3	17.5	0.16	0.685	16.3	18.6	0.60	0.437	16.3	18.0	0.67	0.412
**Valid days**	6.4 ± 0.05	6.0 ± 0.09	15.09	<0.001	6.4 ± 0.04	5.9 ± 0.09	25.77	<0.001	6.4 ± 0.03	6.0 ± 0.06	35.56	<0.001
**Normal weight**	6.3 ± 0.07	6.1 ± 0.11	2.35	0.125	6.4 ± 0.05	5.8 ± 0.09	33.96	<0.001	6.4 ± 0.04	6.0 ± 0.08	20.00	<0.001
**Overweight**	6.5 ± 0.14	6.0 ± 0.20	4.19	0.041	6.3 ± 0.10	5.9 ± 0.18	3.77	0.052	6.4 ± 0.08	6.0 ± 0.14	6.15	0.013
**Obese**	6.6 ± 0.09	5.9 ± 0.16	14.54	<0.001	6.3 ± 0.12	5.7 ± 0.15	9.76	0.002	6.4 ± 0.08	5.8 ± 0.11	19.46	<0.001
**Waking wear time (minutes/day)**	888.5 ± 3.6	832.6 ± 6.4	57.95	<0.001	881.4 ± 2.9	811.2 ± 7.1	83.78	<0.001	884.4 ± 2.2	822.6 ± 4.3	163.71	<0.001
- **Wear during waking periods**												
**Normal weight**	887.6 ± 4.4	834.5 ± 8.8	29.13	<0.001	875.7 ± 3.5	811.0 ± 10.8	32.48	<0.001	880.8 ± 2.8	824.2 ± 6.6	62.33	<0.001
**Overweight**	891.6 ± 7.9	827.3 ± 9.3	27.77	<0.001	885.0 ± 6.1	808.1 ± 9.9	43.73	<0.001	887.3 ± 4.8	817.2 ± 7.7	59.69	<0.001
**Obese**	889.0 ± 10.1	826.0 ± 12.8	14.93	<0.001	898.6 ± 7.9	811.7 ± 10.1	45.93	< 0.001	894.6 ± 6.2	819.1 ± 8.9	48.45	< 0.001
**Total wear time (minutes/day)**	1358.7 ± 6.2	N/A	-	-	1355.8 ± 5.8	N/A	-	-	1357.0 ± 4.2	N/A	-	-

*Note*. Data presented as M ± SE (except for frequencies and percentage values). N/A represents an incalculable value as NHANES employed a waking-hours protocol, not a 24-hour protocol. Dashes (−) indicate the absence of a statistical comparison. *Height data missing for 2 NHANES participants (1 boy and 1 girl). ^†^Probability (*p*) value corresponding to Cochran’s Q-Test for Heterogeneity evaluated at 1 degree of freedom.

## Discussion

Low daily accelerometer wear time threatens validity since there is always the potential for missing data that are not random [2]. Although the valid assessment of sedentary time appears to be most sensitive to low wear time compliance [13], it is clear that most intensities of physical activity may also be underestimated [14]. As a result, interpretation of objectively monitored physical activity using accelerometers has been continually subjected to scrutiny of wear time compliance [2-4]. To address this notable weakness, algorithms have been produced to classify wearing vs. non-wear time [15], and thresholds for minimal wear time have evolved to identify valid data and guide researchers’ analyses [3,14,16]. Despite development of these recommended processes, there continues to be an interest in maximizing wear time compliance during data collection, prior to the data treatment and analysis stages.

In ISCOLE, we chose to ask participants (in this case, 9–11 year old children) to simply wear their waist-worn device continually (i.e., a 24-hour protocol). We demonstrated that a 24-hour protocol produced, on average, more than an hour of additional waist-worn accelerometer waking wear time in 9–11 year old U.S. children compared to a waking-hours protocol using the same device and body attachment site in a sample of similarly aged U.S. children. Wear time characteristics were consistently higher in all ISCOLE country study sites employing a 24-hour protocol compared to those obtained from the NHANES waking-hours protocol. Further, we extended the overall wear time in all ISCOLE country study sites to include non-waking-hours and therefore uniquely and effectively captured movement/non-movement data during the TSET [11] for consistently more than 22 hours of wear time over a 24-hour monitoring period. Troiano et al. [17] recently revealed that the 2011–2012 NHANES cycle achieved a median wear time of approximately 22 hours/day by implementing a 24-hour wrist-worn accelerometer (in a population sample that included adults as well as children). For comparison purposes using the same descriptive statistic (median), these U.S. ISCOLE children provided a median wear time of 23.3 hours/day. An earlier analysis [16] of the 2003–2005 NHANES waking-hours waist-worn data indicated that 6–11 year old children had the lowest mean wear time (13.7 hours/day) compared with the highest of (14.5 hours/day) for 40–49 year old adults, so the very high results for mean wear time in both the 24-hour period (22.6 hours/day) and the waking-hours period (14.8 hours/day) in these ISCOLE children is notable.

Other researchers have attempted to extend wear time compliance using wrist-worn monitors assuming that this attachment site is more convenient and/or comfortable for participants [6]. Wearing time from a 24-hour wrist worn protocol is indeed longer than that obtained from a waking-hours waist-worn protocol, however, it is difficult to discern whether the improvement results from the attachment site (waist versus hip) or the length of the wearing protocol (waking day versus 24-hour) if both attachment site and instructed wear time differ between conditions, as is being implemented in current NHANES data collection cycles [6]. It remains possible that at least some of the observed difference in wear time compliance between the U.S. children studied in ISCOLE and NHANES was due to differential implementation of compliance enhancing strategies. Specifically, the U.S. ISCOLE site conducted reminder phone calls and gave participating children small incentives (e.g., erasers, stickers) for wearing compliance. However, the consistently higher wear time apparent with implementation of the 24-hour accelerometer protocol across all ISCOLE sites (some of which did not visit schools, provide small incentives, or conduct phone calls) relative to the NHANES enhances confidence in our conclusion that merely extending the duration of a waist-worn accelerometer protocol enhances wear time compliance. It is important to point out, however, that ISCOLE accelerometers were distributed in a school setting and multiple children within the same peer group were simultaneously assessed. In contrast, NHANES distributed accelerometers to individual children at a single face-to-face testing center encounter, data collection staff were available only by phone to answer questions or concerns, a reminder post card was mailed to encourage return of the accelerometer, and a monetary incentive was provided for its return. A carefully planned prospective study focused only on the impact of different accelerometer wear time protocol requirements on actual wear time compliance is warranted to rule out competing explanations.

Although the wrist attachment is popular with sleep researchers, waist-worn devices can provide similar estimates of total sleep time as wrist-worn devices [18]. Wrist-worn devices enable extraction of wrist and arm posture which may inform pattern recognition [19], however, they do not appear to perform as well as the waist attachment in terms of estimated energy expenditure [20,5], time spent in moderate-to-vigorous physical activity [5], step-determined physical activity [21], and sedentary time [5]. The researchers involved in the ISCOLE study prioritized these particular movement/non-movement data and believed they could extend participants’ daily wear time (while also capturing their TSET, the subject of a separate analysis [11]) and therefore remained committed to collecting accelerometer data using the waist attachment site.

This was a secondary analysis comparing accelerometer wear time estimates from two distinct cross-sectional studies (ISCOLE and NHANES), both executed in similarly aged free-living samples. There was no external criterion reference for wear time. We merely compared and reported wear time characteristics from both studies using similar data treatment methods. Due to divergent sampling strategies employed for ISCOLE and NHANES we were purposively conservative in our decision to apply inferential analyses using a meta-analytic approach and focusing only on the U.S. ISCOLE vs. the U.S. NHANES data. However, the consistency of extended wear time compliance in all ISCOLE country study sites relative to NHANES is apparent from Figures 1 and 2. This is a particularly salient point because many of the other sites did not implement the same additional compliance strategies that the U.S. site did (indicated in Table 1). The results of this analysis may not be generalized to other age groups. We disclose in detail the ISCOLE accelerometer data collection, management, and treatment manual in Additional file 1 and similar information is publically available for the NHANES data set. Future research should continue to report wear time data obtained from more diverse samples.

## Conclusions

Implementation of a 24-hour waist-worn accelerometry protocol with 9–11 year old U.S. children produced 22.6 out of 24 hours of possible wear time, and 61.8 minutes/day more of waking wear time than the 2003–2006 NHANES’ similarly implemented and processed waking wear time waist-worn accelerometry protocol. Consistent results were obtained internationally. The extended 24-hour protocol may produce an important increase in wear time compliance and, although the focus of a separate analysis [10], also provides an opportunity to study the TSET separate and distinct from physical activity and sedentary time detected during waking-hours. With the emergence of even more water-proof devices (regardless of body attachment site), it may be possible to close this small gap in wear time even further if they can be dried sufficiently following submersion to facilitate comfortable wear, although their real impact on estimates of time spent in physical activity and sedentary behavior remains to be seen.

## Additional file

Additional file 1:
**Manual of procedures for the collection, management, and treatment of accelerometer data in the International Study of Childhood Obesity, Lifestyle and the Environment (ISCOLE).**

## Abbreviations

IRB: Institutional review board
ISCOLE: International study of childhood obesity, lifestyle and the environment
USA: United States of America
NHANES: National health and nutrition examination survey
PACK: PArticipant checKlist

## References

[1] Katzmarzyk PT, Barreira TV, Broyles ST, Champagne CM, Chaput J-P, Fogelholm M (2013). The International Study of Childhood Obesity, Lifestyle and the Environment (ISCOLE): design and methods. BMC Public Health.

[2] Tudor-Locke C, Johnson WD, Katzmarzyk PT (2011). U.S. population profile of time-stamped accelerometer outputs: impact of wear time. J Phys Act Health.

[3] Herrmann SD, Barreira TV, Kang M, Ainsworth BE (2012). How many hours are enough? Accelerometer wear time may provide bias in daily activity estimates. J Phys Act Health.

[4] Matthews CE, Hagstromer M, Pober DM, Bowles HR (2012). Best practices for using physical activity monitors in population-based research. Med Sci Sports Exerc.

[5] Rosenberger ME, Haskell WL, Albinali F, Mota S, Nawyn J, Intille S (2013). Estimating activity and sedentary behavior from an accelerometer on the hip or wrist. Med Sci Sports Exerc.

[6] Choi L, Ward SC, Schnelle JF, Buchowski MS (2012). Assessment of wear/nonwear time classification algorithms for triaxial accelerometer. Med Sci Sports Exerc.

[7] Tudor-Locke C, Camhi SM, Troiano RP (2012). A catalog of rules, variables, and definitions applied to accelerometer data in the National Health and Nutrition Examination Survey, 2003–2006. Prev Chronic Dis..

[8] Tudor-Locke C, Johnson WD, Katzmarzyk PT (2009). Accelerometer-determined steps per day in US adults. Med Sci Sports Exerc.

[9] Scholle S, Beyer U, Bernhard M, Eichholz S, Erler T, Graness P (2011). Normative values of polysomnographic parameters in childhood and adolescence: quantitative sleep parameters. Sleep Med.

[10] Tudor-Locke C, Barreira TV, Schuna JM, Mire EF, Katzmarzyk PT (2014). Fully automated waist-worn accelerometer algorithm for detecting children’s sleep period time separate from 24-hour physical activity or sedentary behaviors. Appl Physiol Nutr Metabol.

[11] Barreira TV, Schuna JM, Jr., Mire EF, Katzmarzyk PT, Chaput JP, Leduc G et al. Identifying children’s nocturnal sleep using 24-hour waist accelerometry. Med Sci Sports Exerc. in press. 10.1249/MSS.0000000000000486 25202840

[12] Mark AE, Janssen I (2008). Dose–response relation between physical activity and blood pressure in youth. Med Sci Sports Exerc.

[13] Masse LC, Fuemmeler BF, Anderson CB, Matthews CE, Trost SG, Catellier DJ (2005). Accelerometer data reduction: a comparison of four reduction algorithms on select outcome variables. Med Sci Sports Exerc.

[14] Herrmann SD, Barreira TV, Kang M, Ainsworth BE. Impact of accelerometer wear time on physical activity data: a NHANES semisimulation data approach. Br J Sports Med. 2012. doi:10.1136/bjsports-2012-091410 10.1136/bjsports-2012-091410 22936409

[15] Choi L, Liu Z, Matthews CE, Buchowski MS (2011). Validation of accelerometer wear and nonwear time classification algorithm. Med Sci Sports Exerc.

[16] Troiano RP, Berrigan D, Dodd KW, Masse LC, Tilert T, McDowell M (2008). Physical activity in the United States measured by accelerometer. Med Sci Sports Exerc.

[17] Troiano RP, McClain JJ, Brychta RJ, Chen KY (2014). Evolution of accelerometer methods for physical activity research. Br J Sports Med.

[18] Kinder JR, Lee KA, Thompson H, Hicks K, Topp K, Madsen KA (2012). Validation of a hip-worn accelerometer in measuring sleep time in children. J Pediatr Nurs.

[19] Rowlands AV, Olds TS, Hillsdon M, Pulsford R, Hurst TL, Eston RG et al. Assessing sedentary behavior with the GENEActiv: introducing the sedentary sphere. Med Sci Sports Exerc. 2013. doi:10.1249/MSS.0000000000000224. 10.1249/MSS.0000000000000224 24263980

[20] Swartz AM, Strath SJ, Bassett DR, O’Brien WL, King GA, Ainsworth BE (2000). Estimation of energy expenditure using CSA accelerometers at hip and wrist sites. Med Sci Sports Exerc.

[21] Tudor-Locke C, Barreira TV, Schuna JM, Jr. Comparison of step outputs for waist and wrist accelerometer attachment sites. Med Sci Sports Exerc. in press. doi:10.1249/MSS.0000000000000476. 10.1249/MSS.0000000000000476 25121517

