# Supplementary material for: Improving wear time compliance with a 24-hour waist-worn accelerometer protocol in the International Study of Childhood Obesity, Lifestyle and the Environment (ISCOLE)
Source: Int J Behav Nutr Phys Act. 2015 Feb 11;12:11. doi: 10.1186/s12966-015-0172-x (PMC4328595; doi:10.1186/s12966-015-0172-x)
Supplement: Additional file 1: — Manual of procedures for the collection, management, and treatment of accelerometer data in the International Study of Childhood Obesity, Lifestyle and the Environment (ISCOLE). [file 12966_2015_172_MOESM1_ESM.pdf]

Manual of procedures for the collection, management, and treatment of accelerometer data in the International Study of Childhood Obesity, Lifestyle and the Environment (ISCOLE)

Contents

Background ... 2

Data Collection ... 2

Choice of instrument ... 2

Protocol ... 4

Instrument initialization ... 4

Instrument wear regimen ... 5

Accelerometer wear instruction ... 5

Compliance enhancing strategies ... 6

Data download ... 6

Immediate determination of valid data ... 7

Participant checklist (PACK) ... 7

Data transfer ... 8

Data Management ... 8

Visual quality control checks ... 8

SAS dataset creation ... 9

Automated quality control checks ... 9

Creation of final data sets ... 10

Data organization ... 10

Initialization errors ... 11

Additional cleaning ... 11

Finalization ... 12

Data Treatment 12
Total Sleep Episode Time (TSET) 12
Non-wear time 15
Wake/wear time 16
Derived variables 16
References 18
Table 1. Accelerometer-derived TSET-, physical activity-, and sedentary behavior-related variables and cut point definitions used in ISCOLE. 23
Contributing Authors 27

Background

The International Study of Childhood Obesity, Lifestyle and the Environment (ISCOLE) is a multi-national cross-sectional study of lifestyle and environmental factors that may influence children's obesity. Data, including objectively monitored physical activity using the ActiGraph GT3X+ accelerometer (ActiGraph LLC, Pensacola, FL, USA), were collected from over 500 children (targeting a mean age of 10 years) in each of the following 12 countries: Australia, Brazil, Canada, China, Colombia, Finland, India, Kenya, Portugal, South Africa, the United Kingdom, and the United States of America. The purpose of this manual of procedures is to provide a detailed description of the ISCOLE study's accelerometer data collection methods, including the management and treatment of the data. Our intent is to provide a clear presentation and rationale for the protocol, decision rules, derived variables, and variable definitions to guide future use of these important objectively monitored data. It is anticipated that these detailed methods will promote the harmonization of accelerometer data collection, management, and treatment across studies, while provoking further research in this area.

Data Collection

Choice of instrument

There are many commercially available research-grade accelerometers that have been used in clinical and population research. Recommendations guiding instrument selection in children's research have previously been published [1], however, only a small number of accelerometers were presented for comparison purposes. Upon inception of the ISCOLE planning process, we conducted a systematic evaluation of each instrument's unique features, advantages, and disadvantages. Based on our own collective experiences and in consultation with established accelerometer experts, we considered six potential accelerometers and multi-sensor

devices for use in ISCOLE: 1) the GT3X (ActiGraph, LLC, Pensacola, Florida, USA), 2) the GT3X+ (ActiGraph, LLC, Pensacola, Florida, USA), 3) the ActivPAL (Pal Tech Ltd., Glasgow, United Kingdom), 4) the Actical (Philips Respironics, Mini Mitter Company Inc., Oregon, USA), 5) the Actiheart (CamNtech Ltd., Cambridge, UK) and 6) the SenseWear Armband (Bodymedia, Pittsburg, Pennsylvania, USA).

The factors that were considered included memory capacity, data collection time interval options (epoch length), validity and reliability evidence in children, price, technical support, and safety (among others). Expert consultations reinforced the importance of collecting raw acceleration data to assure maximum utility well into the future with the potential additional utility for pattern recognition. This requirement alone eliminated most of the devices considered. The final two devices remaining in consideration for use in ISCOLE were the ActiGraph GT3X+ and the ActivPAL. We chose the Actigraph GT3X+ for the following reasons: 1) data could be distilled to infer time in moderate-to-vigorous intensity physical activity using evidence-based cut points (a similar process did not exist for the ActivPAL at the time); 2) there was comparatively more published literature about previous generations of ActiGraph devices, especially with regard to reliability and validity of physical activity measurement in children [2, 3] (we could not locate anything about the ActivPAL and children prior to 2010); 3) our pilot (unpublished) data suggested that participants may experience difficulties keeping the ActivPAL fixed to their thigh for multiple 24-hour days whereas this was not the case with the waist-worn ActiGraph; and 4) a minimum of 840 units were needed (70 for each country) in a short time frame, and ActiGraph LLC was able to assure prompt delivery of this purchased quota.

Protocol

Instrument initialization

Accelerometers were fully charged prior to initialization. Initialization is the process by which accelerometers are prepared for data collection, including setting data collection start and stop dates and times, selecting optional instrument features (e.g., data sampling rates), and deselecting default settings that may not be desired.

Version 5.6 (or higher, as new releases were provided) of the ActiLife software (ActiGraph LLC, Pensacola, Florida, USA) was used to initialize the devices. The GT3X+ accelerometer can collect raw acceleration data at sampling rates between 30 and 100 Hertz (Hz); however, at the time of purchase, the internal memory onboard the GT3X+ (256 mb) allowed for a maximum sampling rate of 80 Hz in order to collect data for at least 7 days. Due to this original constraint, an 80 Hz sampling rate was selected for all countries involved in ISCOLE. Midway through the ISCOLE data collection (June 2012), ActiGraph LLC added a new feature in Version 6.2.1 called the "idle sleep mode." This feature was intended to save battery life when no movement was detected. Once activated, the idle sleep mode prevented full recording of the raw data signal. Because of this, and as we were interested in capturing 24-hour data (see below), the ISCOLE protocol called for this newly added default feature to be disabled during initialization. The accelerometers were set to begin data collection at midnight immediately following the first day that the children received the devices. The first day of accelerometer distribution was not considered a full day of wear, so data collection during this time was not necessary. Nevertheless, the children were instructed to wear the device from time of distribution and they were not aware of the initialization start time. A stop time was not selected.

Instrument wear regimen

Historically, calibration of the ActiGraph accelerometer was based on devices worn at the hip location among adults [4]. Subsequent calibration studies in children aimed at estimating time in different intensities of activity (sedentary, light, moderate, and vigorous intensities) from activity counts were also conducted with the accelerometer worn at the hip [5-9]. Despite recent interest in using wrist worn accelerometers to measure physical activity and sedentary behavior, no published studies among children have validated the ActiGraph accelerometer for estimating time in different intensities of activity when worn at the wrist. Hence, we chose to remain consistent with methodologies from pediatric calibration studies [5-9] by instructing children to wear the ActiGraph at the hip location. Specifically, the accelerometer was attached to the participant using an elastic belt worn around the waist with an adjustable clip. The accelerometer unit itself was placed in line with the mid-axillary line and lying on the iliac crest (i.e., hip location) on the right side of the body.

Participants were asked to wear the device for 24 hours/day for 7 consecutive full days (not including the initial familiarization period of the first day and the morning of the final day before accelerometer retrieval), including 2 weekend days. Participants were instructed to remove the accelerometer for water-based activities (i.e., showering, swimming). The 24-hour protocol was selected in an attempt to increase wear time compliance [10, 11] and also to obtain information about children's total sleep episode time (TSET) separate and distinct from physical activity and sedentary behaviors detected during waking hours [12].

Accelerometer wear instruction

Accelerometers were distributed in a school classroom setting. A standardized set of instructions was read aloud to the assembled class of participants, and individually again as

required. Additionally a hard-copy paper version of the instructions, a picture demonstrating correct attachment, and contact information for queries was given to each participant to take home. All documents/forms were translated and back-translated, as necessary, into each study site's local language following approved procedures of the local institutions. The children were individually fitted with the accelerometer and asked to remove and re-attach the belt, demonstrating appropriate placement and orientation of the accelerometer. The children were able to ask questions during these instructions.

Compliance enhancing strategies

Several behavioral strategies were implemented in an attempt to maximize compliance to the wear regimen. Strategies included any combination of the following: up to two phone calls to parents (one during the weekday and one during the weekend); daily visits to schools by local study staff to inspect whether the accelerometer was being worn correctly; inspection/reporting by the school teacher; and if allowed by local Institutional Review Board (IRB)/ Ethics Committee rules and regulations, distribution of small daily incentives (e.g., erasers, stickers) for correctly wearing the accelerometer. Local sites also had the option (again, with local IRB/Ethics Committee approval) to ask children to wear the device a second week if they did not accumulate sufficient wear time the first week to provide valid data (see below for a definition), or if the accelerometer had malfunctioned in some way.

Data download

ActiLife version 5.6 (or higher) software was used to download recorded data immediately upon retrieval of each accelerometer. The downloading process produced an .AGD file with the following settings: 1 second epoch, 3 axes of orientation, steps, lux (ambient light), inclinometer, and low frequency extension (LFE) filter. The 1 second epoch was selected to offer

the greatest flexibility in terms of later reintegration into longer epochs. The ActiGraph GT3X+'s acceleration signal was processed using the LFE filter to best approximate activity count outputs from a previous generation of ActiGraph (model 7164)[13] used in pediatric calibration studies identifying cut points for physical activity and sedentary behavior [5, 9]. This decision was also made to facilitate comparisons with data obtained from the National Health and Nutrition Examination Survey (NHANES 2003-2006) [14]. However, step count data were later reprocessed using the default filter because the LFE filter is known to produce a large over-estimation of step counts [13, 15]. The file name created during the process included the participant ID, a country ID, part of the individual accelerometer's serial number, and code indicating whether it was the first time or second time that the child wore the accelerometer.

Immediate determination of valid data

Immediately following data download, the ActiLife software was used to determine whether valid data were obtained during the period of wear. Specifically, local study staff checked whether participants had at least 4 days of data, including 1 weekend day with greater than 10 hours/day of wear time using the ActiLife software's identified "old daily algorithm." As already alluded to, local sites had the option to ask participants to wear an accelerometer for a second week if there were insufficient data collected, and if there was prior local IRB approval of this option. The decision to collect additional weeks of monitoring was made on a local and case-by-case basis.

Participant checklist (PACK)

Local sites were required to complete a PArticipant ChecKlist (PACK) for every participant to track their progress through all aspects of the ISCOLE study, including accelerometry data collection. Specifically, the PACK included prompts to record accelerometer

ID numbers along with distribution and retrieval dates. It also served as a record of compliance checks, the number of valid days identified during data download, and whether the child was asked to wear an accelerometer for a second week (identified as an “additional monitoring file.”) All data collected on paper forms of the PACK were electronically entered by local study staff into the ISCOLE study Coordinating Center’s secure website.

Data transfer

After data download was completed and data were deemed valid, a compressed (.ZIP) 1 sec .AGD file was transferred via upload to the study Coordinating Center’s secure website where it could be linked to the associated electronically-entered PACK. Files were systematically matched with the correct participant ID codes. If a mismatch occurred, an automatic error message would appear and the uploading process was blocked until a correction was made. An identifier with date and time was added to every file during the uploading process. Completion of this final step signified successful transfer of data from the local data collection site to the study Coordinating Center. However, as two additional back-up processes, each country sent their de-identified data to the Coordinating Center on an external hard-drive and also secured and retained the original raw data locally.

Data Management

Upon receipt of the accelerometer files, the ISCOLE staff at the coordinating center conducted a series of data checks before the data were treated and summarized.

Visual quality control checks

A trained data manager conducted thorough data queries to ensure that protocols were adhered to and that quality data were collected at each site. The uploaded files were decompressed and data checks were performed on the 1 second .AGD files. These initial queries

included verifying file names to determine compliance with the study protocol, confirming that file sizes were within acceptable ranges, and cross-referencing PACK items to confirm that all uploaded files were correctly identified as valid. Any discrepancies noted that could not be resolved through data management triggered communication with the local data collection site to resolve the problem, including re-uploading individual files as necessary.

SAS dataset creation

Once the visual checks were completed, all files from each country were re-integrated into 60 second epochs for the creation of two SAS datasets to be utilized in further quality control checks. The .AGD files (individual SQLite databases) from each country were then read into a Microsoft SQL Server database to allow for efficient data storage. All accelerometry data for each country from the Microsoft SQL Server database were imported into SAS and two country-specific datasets were created. The first dataset, named "Header," contained information downloaded directly from the device including the serial number of the device and a date/time stamp indicating when it was initialized and also when the data were downloaded. The second dataset included minute-by-minute data obtained from the accelerometer which included, among other variables, the activity counts for each axis and steps.

Automated quality control checks

Using the Header file, SAS syntax was prepared to automatically compare data collection start time and the serial number of accelerometers linked to each participant to determine if files had been incorrectly named or if a second participant had worn the accelerometer without re-initialization. The serial number of the Header was also compared to the serial number contained in the file name; if a mismatch was detected, the data collection site was contacted for clarification. In addition, the participants' identification numbers from the PACK were then

compared to those in the accelerometry data to show if there were any accelerometer files that should have been uploaded but were apparently missing, or if there were accelerometer data for participants that were not indicated on the PACK. A similar automated check was performed to ensure that any participant requiring additional monitoring had the proper data stored (additional monitoring was identified and the initial monitoring data file was not included). Discrepancies (i.e., missing expected .AGD files) were further investigated, including communicating with the local sites to resolve identified issues and/or ascertain missing data.

Creation of final data sets

Once all the initial quality control checks were finalized and each participant had a unique and correctly identified file, three final SAS datasets were created. The main dataset contained data in 60 second epochs (derived using the LFE filter), the second dataset contained data in 15 second epochs (also derived using the LFE filter), and the final dataset contained data in 60 second epochs (derived using the default filter; created to extract the step counts).

Data organization

In order to facilitate further data cleaning and analyses, variables were created, renamed and systematically organized. The date/time stamp was separated into two variables and formatted with SAS-specific date and time formats. The four variables associated with the devices' inclinometer measurements were formatted into a single variable with four possible responses (off, standing, sitting or lying). Vector magnitude was calculated as the square root of the sum of the squares of the three axes. The step count data were stripped from the dataset defined by the default filter and used to replace those calculated with the LFE filter.

Initialization errors

Utilizing the main dataset (60 sec LFE filter), the first minute for each participant's data was identified in order to verify that the protocol was followed and all devices were initialized at midnight the day after they were given to participants, as recorded on the PACK. We deleted all data recorded prior to midnight of the indicated day of accelerometer distribution in instances where the device was initialized at any time other than midnight. Because children were only seen during the school week, initialization times were generally expected to occur on Tuesday-Saturday. If it was determined that an accelerometer was initialized on a Sunday or Monday, then the first day of data was visually inspected and compared to the PACK to conclude whether or not the device was being worn at the time of initialization; if not, the first day's data were deleted. The PACK was then referenced to identify any instances in which the first date of data collected was not equal to the day after accelerometer distribution.

Additional cleaning

The last day of data collected for each participant was clarified in order to standardize the files and conduct additional cleaning procedures. We examined any instances in which the last date of data collected was not equal to the date of device return according to the PACK. We isolated cases where the difference in these dates was more than one day, and visually inspected any files indicating that the last day of data collected was within 7 days of the application date according to the PACK. Following this cleaning process, we deleted the last date of data for all participants to ensure that all days used in physical activity analyses contained 24 hours of data. We further visually inspected data from any participants with more than 14 days of data. Such cases could indicate that the files contained valid data for more than one participant in a given file (file naming or download/initialization error), late accelerometer return because the

participant forgot to wear the device or was not present on the distribution day, or simply protocol deviation due to the device not being stopped upon retrieval.

Finalization

Once all queries created in the data management process were properly resolved through at the study coordinating site or via communication with the local collection sites, each recorded minute for each participant was counted and labeled to facilitate analyses. Sequential 24-hour days were identified and labeled separately according to both midnight-to-midnight and noon-to-noon definitions, enabling maximum utility for evaluating both daytime behaviors and TSET (see below) undisrupted by applying conventional calendar day 24-hour clocks. As per protocol and in order to ensure valid and comparable data were used in analyses, any data collected after day 7 were deleted.

Data Treatment

Data treatment included the application of decision rules used to score the data and to derive and define variables. Sequentially, we first identified TSET, non-wear time, and wake/wearing time, before finally producing derived variables representing estimates of sleep-related variables, physical activity, and sedentary behavior. The process is summarized below.

Total Sleep Episode Time (TSET)

Following management, integration, and cleaning of each participant's data into a single minute-by-minute file, we first identified "sleep period time" as an initial step to score the data. We defined sleep period time according to Scholle et al.[16] as "time of sleep onset to the end of sleep, including all sleep epochs and wakefulness after onset." Sleep period time for each participant was determined using a novel and fully-automated algorithm specifically developed for use in ISCOLE and other epidemiological studies employing a 24-hour waist-worn

accelerometer protocol in children [17]. Specifically, we have shown that this algorithm produced sleep period time estimates similar to those obtained with expert visual inspection of accelerometer data [17]. Further refinement of this algorithm made it possible for us to calculate TSET and exclude extended periods of wakefulness during previously determined sleep episode time [12].

The ISCOLE researchers' decision to apply a 24-hour accelerometer protocol creates a distinctive situation which affects the description of time spent sleeping and in different intensities of physical activity (when awake). For example, sleep is most often expressed in terms of nocturnal sleep (i.e., sleep period time) which, in most cases, corresponds to behavior spanning two calendar days: beginning one day (e.g., 9:00 p.m.) and ending the next (e.g., 7:00 a.m.). Therefore, we utilized a noon-to-noon time frame to determine TSET. Physical activity and sedentary behavior data, on the other hand, are usually expressed as daily averages or total time based on a calendar day (12:00 a.m. – 11:59 p.m.). When estimating physical activity and sedentary behavior with a waking-time protocol, in which participants wear the accelerometer only during awake periods, the amount of time spent in different intensities of physical activity and sedentary behavior is summed and the remaining time within a 24-hour day is classified as non-wear. While the 24-hour wear protocol used in ISCOLE does make it possible to classify residual minutes as sleep or non-wear, it is not entirely appropriate to simply classify them as TSET. In most cases, two disjointed sleep periods would have to be considered separately using a calendar day approach to data analysis – the sleep period running from midnight to morning wake time and the sleep period beginning in the evening and lasting until midnight. Data processing revealed that TSET, non-wear, and physical activity time should not be totaled to

describe a 24-hour day. We therefore created separate data sets for TSET and for physical activity-related variables before combining these derived variables into a final locked data set.

In order to accommodate the issues of sleep outside of a calendar day, each minute was given a probability of sleep as determined by Sadeh's algorithm [18], and a binary indicator variable was assigned to identify each minute as sleep (1) or wake (0). Minutes previously defined as wake by the Sadeh algorithm but determined to be in the "off" position by the accelerometer's inclinometer were re-defined as sleep. We then used a separate algorithm adapted from SAS code developed by the National Cancer Institute (http://riskfactor.cancer.gov/tools/nhanes_pam/create.html) to determine sleep period time and TSET for each noon-to-noon (12:00 a.m. to 11:59 p.m.) day. The beginning of a sleep episode (nocturnal sleep onset) was identified as the first 5 consecutive minutes of sleep, and the end of a sleep episode (nocturnal sleep offset) was identified as the first 10 or 20 consecutive minutes of wake time, depending on the time of day (10 minutes – 5:00 a.m. to 11:58 a.m.; 20 minutes – 7:00 p.m. to 4:59 a.m.). A sleep episode was only identified when at least 160 minutes had elapsed following nocturnal sleep onset and could contain an unlimited number of non-consecutive wake minutes. Multiple sleep episodes (≥ 160 minutes) were allowed during each 24-hour noon-to-noon day, but only sleep episodes that began between 7:00 p.m. and 5:59 a.m. were considered. If sleep episodes were separated by less than 20 minutes, then they were combined so that the first minute of the first sleep episode through to the final minute of the last sleep episode constituted a single sleep episode. Sleep episodes that were separated by at least 20 minutes were not combined. The decision to consider the 20 minute separation was based on close examination of the data which revealed that computation of rolling averages (across minutes) meant that 2-3 minutes of low to moderate activity (265-400 activity counts/minute) in

a 10 minute period (with all other epochs having 0 activity counts/minute) could lead to 8-15 minute periods being classified as waking time. Twenty minutes was also found to be at the lower end of alternative time frames considered that resulted in most participants having only one sleep episode.

A second algorithm was then used to identify periods of non-wear within a previously defined sleep episode. Exploratory analysis revealed that commonly used non-wear algorithms relying on shorter time durations of consecutive minutes registering 0 activity counts (e.g., ≥ 20 or ≥ 60 minutes) were overly sensitive and classified non-wear time in nearly every sleep episode. Non-wear identification within the sleep episode was improved by lengthening the minimum time duration of consecutive 0 activity counts needed to define a non-wear period (e.g., ≥ 90 minutes). Therefore, we identified non-wear within the sleep episode. Non-wear was identified when 90 consecutive minutes of 0 activity counts were encountered while allowing for up to 2 minutes of non-zero activity counts. The non-wear period ended when a third minute of non-zero activity counts was reached. If at least 90% of a sleep episode consisted of non-wear, then all minutes within that sleep episode were redefined as non-wear instead of sleep. TSET in minutes/day was calculated as the aggregate time between the start and end of each sleep episode.

Non-wear time

A separate non-wear algorithm was run on all remaining minutes not identified as part of the TSET in previous data processing. Non-wear periods were defined as any sequence of at least 20 consecutive minutes of 0 activity counts [19]. Each non-wear period ended with any minute of non-zero activity counts. Minutes between the start and end of each non-wear period were labeled as non-wear, and total non-wear (minutes/day) was calculated as the aggregate time spent

in all non-wear periods within a midnight-to-midnight day (12:00 a.m. to 11:59 p.m.), including non-wear detected during the TSET.

Wake/wear time

Remaining epochs in the minute-by-minute accelerometry data file not identified as part of the TSET or non-wear were labeled as wake/wearing time. Minutes with implausibly high activity count values (≥ 20,000 activity counts/minute) were examined to determine if they occurred during the TSET or during waking hours, and redefined as a minute of TSET or non-wear accordingly [20]. We considered imputing these data points by averaging surrounding values. However, upon closer inspection it was apparent that these adjacent minutes were also oddly high, just not above the designated threshold, and we decided it was prudent not to do this as it would result in minutes likely being misclassified as MVPA.

Derived variables

Following the identification of the TSET, non-wear, and wake/wearing time, activity count and step values for each epoch were scored and used to derive a number of variables, catalogued in Table 1. The TSET and non-wear identifier variables were merged with the 15 second epoch file by participant identifier, date, and time in order to facilitate the identification and summary of the physical activity and sedentary behavior variables.

Weekly averages for all TSET-related variables were calculated using only noon-to-noon days where valid TSET was accumulated (TSET > 0) and only for participants with at least 3 nights of valid TSET, including one weekend night. Separate TSET-related averages were also computed for the following categories: 1) school night bedtime and school day wake time (Monday night-Thursday morning), 2) school night bedtime and weekend wake time (Friday

night-Saturday morning), 3) weekend bedtime and weekend wake time (Saturday night-Sunday morning), and 4) weekend bedtime and school day wake time (Sunday night-Monday morning).

Weekly averages for each activity count and step-based variable (excluding TSET variables) were calculated using only minutes for which the participant was awake and wearing the device on valid midnight-to-midnight days (at least 10 hours of wake/wear time in a 24-hour period)[14] and only for participants with at least 4 valid days (including one weekend day). One weekend day was required because of the known differences in activity levels between weekdays and weekend days in children [21-23] and because it has been catalogued as a frequently used analytical choice applied previously to NHANES children's and adolescents' accelerometer data [24]. Physical activity and sedentary behavior variables were derived using activity count cut points suggested by Treuth et al. [9] to allow comparison of results with the largest multi-national pediatric physical activity study [25]. Moreover, we also quantified physical activity and sedentary behavior using the Evenson et al. [5] activity count cut points as these have been shown to maximize activity intensity classification accuracy when compared to other pediatric activity count cut points [26]. Although recent interest has developed in using multiple features extracted from the accelerometer's raw signal to classify activity type and estimate energy expenditure [27], no consensus or best practices for using such methodologies with pediatric populations exist at this time. In addition to the weekly summary, all physical activity and sedentary behavior estimates were averaged separately for weekdays and weekend days, using only waking time and wearing minutes from valid days for participants with at least 4 valid days (including one weekend day).

References

1. McClain JJ, Tudor-Locke C: **Objective monitoring of physical activity in children: considerations for instrument selection.** *J Sci Med Sport* 2008, **12:**526-533.
2. De Vries SI, Van Hirtum HWJ, Bakker I, Hopman-Rock M, Hirasing RA, Van Mechelen W: **Validity and reproducibility of motion sensors in youth: a systematic update.** *Med Sci Sports Exerc* 2009, **41:**818-827.
3. Mattocks C, Ness A, Leary S, Tilling K, Blair SN, Shield J, Deere K, Saunders J, Kirkby J, Smith GD, et al: **Use of accelerometers in a large field-based study of children: protocols, design issues, and effects on precision.** *Journal of Physical Activity & Health* 2008, **5 Suppl 1:**S98-111.
4. Freedson PS, Melanson E, Sirard J: **Calibration of the Computer Science and Applications, Inc. accelerometer.** *Med Sci Sports Exerc* 1998, **30:**777-781.
5. Evenson KR, Catellier DJ, Gill K, Ondrak KS, McMurray RG: **Calibration of two objective measures of physical activity for children.** *J Sports Sci* 2008, **26:**1557-1565.
6. Freedson P, Pober D, Janz KF: **Calibration of accelerometer output for children.** *Med Sci Sports Exerc* 2005, **37:**S523-530.
7. Mattocks C, Leary S, Ness A, Deere K, Saunders J, Tilling K, Kirkby J, Blair SN, Riddoch C: **Calibration of an accelerometer during free-living activities in children.** *Int J Pediatr Obes* 2007, **2:**218-226.
8. Puyau MR, Adolph AL, Vohra FA, Butte NF: **Validation and calibration of physical activity monitors in children.** *Obes Res* 2002, **10:**150-157.
9. Treuth MS, Schmitz K, Catellier DJ, McMurray RG, Murray DM, Almeida MJ, Going S, Norman JE, Pate R: **Defining accelerometer thresholds for activity intensities in adolescent girls.** *Med Sci Sports Exerc* 2004, **36:**1259-1266.

10. Butte NF, Puyau MR, Adolph AL, Vohra FA, Zakeri I: **Physical activity in nonoverweight and overweight Hispanic children and adolescents.** *Med Sci Sports Exerc* 2007, **39:**1257-1266.

11. Treuth MS, Sherwood NE, Butte NF, McClanahan B, Obarzanek E, Zhou A, Ayers C, Adolph A, Jordan J, Jacobs DR, Rochon J: **Validity and reliability of activity measures in African-American girls for GEMS.** *Med Sci Sports Exerc* 2003, **35:**532-539.

12. Barreira TV, Schuna JM, Jr., Mire EF, Katzmarzyk PT, Chaput JP, Leduc G, Tudor-Locke C: **Identifying children's nocturnal sleep using 24-hour waist accelerometry.** *Med Sci Sports Exerc* in press.

13. Cain KL, Conway TL, Adams MA, Husak LE, Sallis JF: **Comparison of older and newer generations of ActiGraph accelerometers with the normal filter and the low frequency extension.** *Int J Behav Nutr Phys Act* 2013, **10:**51.

14. Troiano RP, Berrigan D, Dodd KW, Masse LC, Tilert T, McDowell M: **Physical activity in the United States measured by accelerometer.** *Med Sci Sports Exerc* 2008, **40:**181-188.

15. Barreira TV, Brouillette RM, Foil HC, Keller JN, Tudor-Locke C: **Comparison of older adults' steps/day using NL-1000 pedometer and two GT3X+ accelerometer filters.** *J Aging Phys Act* 2012, **21:**402-416.

16. Scholle S, Beyer U, Bernhard M, Eichholz S, Erler T, Graness P, Goldmann-Schnalke B, Heisch K, Kirchhoff F, Klementz K, et al: **Normative values of polysomnographic parameters in childhood and adolescence: quantitative sleep parameters.** *Sleep Med* 2011, **12:**542-549.

17. Tudor-Locke C, Barreira TV, Schuna Jr. JM, Mire EF, Katzmarzyk PT: **Fully automated waist-worn accelerometer algorithm for detecting children's sleep period time separate from 24-hour physical activity or sedentary behaviors.** *Applied Physiology and Nutrition Metabolism* 2014, **39:**53-57.

18. Sadeh A, Sharkey KM, Carskadon MA: **Activity-based sleep-wake identification: an empirical test of methodological issues.** *Sleep* 1994, **17:**201-207.

19. Mark AE, Janssen I: **Dose-response relation between physical activity and blood pressure in youth.** *Med Sci Sports Exerc* 2008, **40:**1007-1012.

20. Masse LC, Fuemmeler BF, Anderson CB, Matthews CE, Trost SG, Catellier DJ, Treuth M: **Accelerometer data reduction: a comparison of four reduction algorithms on select outcome variables.** *Med Sci Sports Exerc* 2005, **37:**S544-554.

21. Rowe DA, Mahar MI, Raedeke TD, Lore J: **Measuring physical activity in children with pedometers: Reliability, reactivity, and replacement of missing data.** *Pediatr Exerc Sci* 2004, **16:**343-354.

22. Treuth MS, Catellier DJ, Schmitz KH, Pate RR, Elder JP, McMurray RG, Blew RM, Yang S, Webber L: **Weekend and weekday patterns of physical activity in overweight and normal-weight adolescent girls.** *Obesity (Silver Spring)* 2007, **15:**1782-1788.

23. Rowlands AV, Pilgrim EL, Eston RG: **Patterns of habitual activity across weekdays and weekend days in 9-11-year-old children.** *Prev Med* 2008, **46:**317-324.

24. Tudor-Locke C, Camhi SM, Troiano RP: **A catalog of rules, variables, and definitions applied to accelerometer data in the National Health and Nutrition Examination Survey, 2003-2006.** *Prev Chronic Dis* 2012, **9:**E113.

25. Ekelund U, Luan J, Sherar LB, Esliger DW, Griew P, Cooper A: **Moderate to vigorous physical activity and sedentary time and cardiometabolic risk factors in children and adolescents.** *JAMA* 2012, **307:**704-712.

26. Trost SG, Loprinzi PD, Moore R, Pfeiffer KA: **Comparison of accelerometer cut points for predicting activity intensity in youth.** *Med Sci Sports Exerc* 2011, **43:**1360-1368.

27. Zhang S, Rowlands AV, Murray P, Hurst TL: **Physical activity classification using the GENEA wrist-worn accelerometer.** *Med Sci Sports Exerc* 2012, **44:**742-748.

28. Tudor-Locke C, Ainsworth BE, Thompson RW, Matthews CE: **Comparison of pedometer and accelerometer measures of free-living physical activity.** *Med Sci Sports Exerc* 2002, **34:**2045-2051.

29. Tudor-Locke C, Brashear MM, Johnson WD, Katzmarzyk PT: **Accelerometer profiles of physical activity and inactivity in normal weight, overweight, and obese U.S. men and women.** *Int J Behav Nutr Phys Act* 2010, **7:**60.

30. Tudor-Locke C, Camhi SM, Leonardi C, Johnson WD, Katzmarzyk PT, Earnest CP, Church TS: **Patterns of adults stepping cadence in the 2005-2006 NHANES.** *Prev Med* 2011, **53:**178-181.

31. Tudor-Locke C, Brashear MM, Katzmarzyk PT, Johnson WD: **Peak stepping cadence in free-living adults: 2005-2006 NHANES.** *J Phys Act Health* 2012, **9:**1125-1129.

32. Barreira TV, Katzmarzyk PT, Johnson WD, Tudor-Locke C: **Cadence patterns and peak cadence in U.S. children and adolescents: NHANES 2005-2006.** *Med Sci Sports Exerc* 2012, **44:**1721-1727.

33. Healy GN, Dunstan DW, Salmon J, Cerin E, Shaw JE, Zimmet PZ, Owen N: **Breaks in sedentary time: beneficial associations with metabolic risk.** *Diabetes Care* 2008, **31:**661-666.

Table 1. Accelerometer-derived TSET-, physical activity-, and sedentary behavior-related variables and cut point definitions used in ISCOLE.

Variable	Definitions	References
Sleep-related variables		
Sleep period time	Duration of time from nocturnal sleep onset to nocturnal sleep offset, including all minutes scored as sleep or wake	Based on previously published variable definition [17]
Nocturnal sleep onset	The beginning of a sleep episode (nocturnal sleep onset) was identified as the first 5 consecutive minutes of sleep (12:00 p.m. to 11:59 a.m.)	[12]
Nocturnal sleep offset	The end of a sleep episode (nocturnal sleep offset) was identified within a noon-to-noon day (12:00 p.m. to 11:59 a.m.) as the first 10 or 20 consecutive minutes of wake time, depending on the time of day (10 minutes – 5:00 a.m. to 11:58 a.m.; 20 minutes – 7:00 p.m. to 4:59 a.m.).	[12]
Sleep episode	A sleep episode was only identified when at least 160 minutes had elapsed following nocturnal sleep onset and could contain an unlimited number of non-consecutive wake minutes. Multiple sleep episodes (≥ 160 minutes) were allowed during each 24-hour	[12]

	noon-to-noon day, but only sleep episodes that began between 7:00 p.m. and 5:59 a.m. were considered. If sleep episodes were separated by less than 20 minutes, then they were combined so that the first minute of the first sleep episode through to the final minute of the last sleep episode constituted a single sleep episode. Sleep episodes that were separated by at least 20 minutes were not combined.	
Total sleep episode time	Total minutes from all sleep episodes occurring during the sleep period time	[12]
Activity count metrics		
Activity counts/day	Sum of daily activity counts	[28]
Activity counts/min	Sum of daily activity counts/number of min worn	[28]
Time at different activity intensities – Treuth cutpoints (min/day)		
Sedentary	< 100 activity counts/min	[9]
Light intensity	100-2999 activity counts/min	
Moderate intensity	3000-5200 activity counts/min	
Vigorous intensity	≥ 5201 activity counts/min	
Moderate-to-vigorous intensity	≥ 3000 activity counts/min	
Time at different activity intensities – Evenson cutpoints (min/day)		
Sedentary	< 26 activity counts/15 sec	[5]
Light intensity	26-573 activity counts/15 sec	

Moderate intensity	574-1002 activity counts/15 sec	
Vigorous intensity	≥ 1003 activity counts/15 sec	
Moderate-to-vigorous intensity	≥ 574 activity counts/15 sec	
Step count metrics		
Steps/day	Sum of daily steps	[28]
Steps/min	Sum of daily steps/number of min worn	[29]
Time and steps in incremental cadence bands (min/day and steps/day)		
Non-movement	0 steps/minute during valid wear time	[30]
Incidental movement	1-19 steps/min	
Sporadic movement	20-39 steps/min	
Purposeful steps	40-59 steps/min	
Slow walking	60-79 steps/min	
Medium walking	80-99 steps/min	
Brisk walking	100-119 steps/min	
Faster locomotion	120 steps/min	
Any movement*	> 0 steps/min	
Non-incidental movement*	> 19 steps/min	
Peak cadence indicators (steps/min)		
Peak 1-min cadence	Steps/min recorded for the single highest min in a day	[31]

Peak 30-min cadence	Average steps/min recorded for the 30 highest, but not necessarily consecutive, min in a day	
Peak 60-min cadence	Average steps/min recorded for the 60 highest, but not necessarily consecutive, min in a day	[32]
Breaks in sedentary time (transitions/day)		
Transitions/day	Total occurrences of when activity counts rose from < 100 activity counts in 1 min to ≥ 100 activity counts in the subsequent min	[33]

Contributing Authors

Catrine Tudor-Locke[1] (tudor-locke@pbrc.edu), Tiago V. Barreira[1] (tiago.barreira@pbrc.edu), John M. Schuna Jr. [1] (john.schuna@pbrc.edu), Emily F. Mire[1] (emily.mire@pbrc.edu), Jean-Philippe Chaput[2] (jpchaput@cheo.on.ca), Mikael Fogelholm[3] (mikael.fogelholm@helsinki.fi) Gang Hu[1] (gang.hu@pbrc.edu), Rebecca Kuriyan[4] (rebecca@sjri.res.in), Anura Kurpad[4] (a.kurpad@sjri.res.in), Estelle V. Lambert[5] (vicki.lambert@uct.ac.za), Carol Maher[6] (carol.maher@unisa.edu.au), José Maia[7] (jmaia@fade.up.pt), Victor Matsudo[8] (matsudo@celafiscs.org.br), Tim Olds[6] (timothy.olds@unisa.edu.au), Vincent Onywera[9] (vonywera@gmail.com), Olga L. Sarmiento[10] (osarmien@uniandes.edu.co), Martyn Standage[11] (m.standage@bath.ac.uk), Mark S. Tremblay[2] (mtremblay@cheo.on.ca), Pei Zhao[12] (juliapeizhao@qq.com), Timothy S. Church[1] (timothy.church@pbrc.edu), and Peter T. Katzmarzyk[1] (peter.katzmarzyk@pbrc.edu)

[1]Pennington Biomedical Research Center, Baton Rouge, United States; [2]Children's Hospital of Eastern Ontario Research Institute, Ottawa, Canada; [3]University of Helsinki, Helsinki, Finland; [4]St. Johns Research Institute, Bangalore, India; [5]University of Cape Town, Cape Town, South Africa; [6]University of South Australia, Adelaide, Australia;[7]Faculdade de Desporto, University of Porto, Porto, Portugal; [8]Centro de Estudos do Laboratório de Aptidão Física de São Caetano do Sul (CELAFISCS), Sao Paulo, Brazil; [9]Kenyatta University, Nairobi, Kenya; [10]Universidad de los Andes, Bogota, Colombia; [11]University of Bath, Bath, United Kingdom; [12]Tianjin Women's and Children's Health Center, Tianjin, China.
